# Supplementary material for: The interactome of intact mitochondria by cross-linking mass spectrometry provides evidence for coexisting respiratory supercomplexes
Source: Mol Cell Proteomics. 2017 Dec 8;17(2):216–32. doi: 10.1074/mcp.RA117.000470 (PMC5795388; doi:10.1074/mcp.RA117.000470)
Supplement: Supplemental Data [file supp_RA117.000470_133922_0_supp_23978_fzffwf.zip › spectra_annotation/mito_DR_spectra_annotation/486-1-5-1-5-1.pdf]

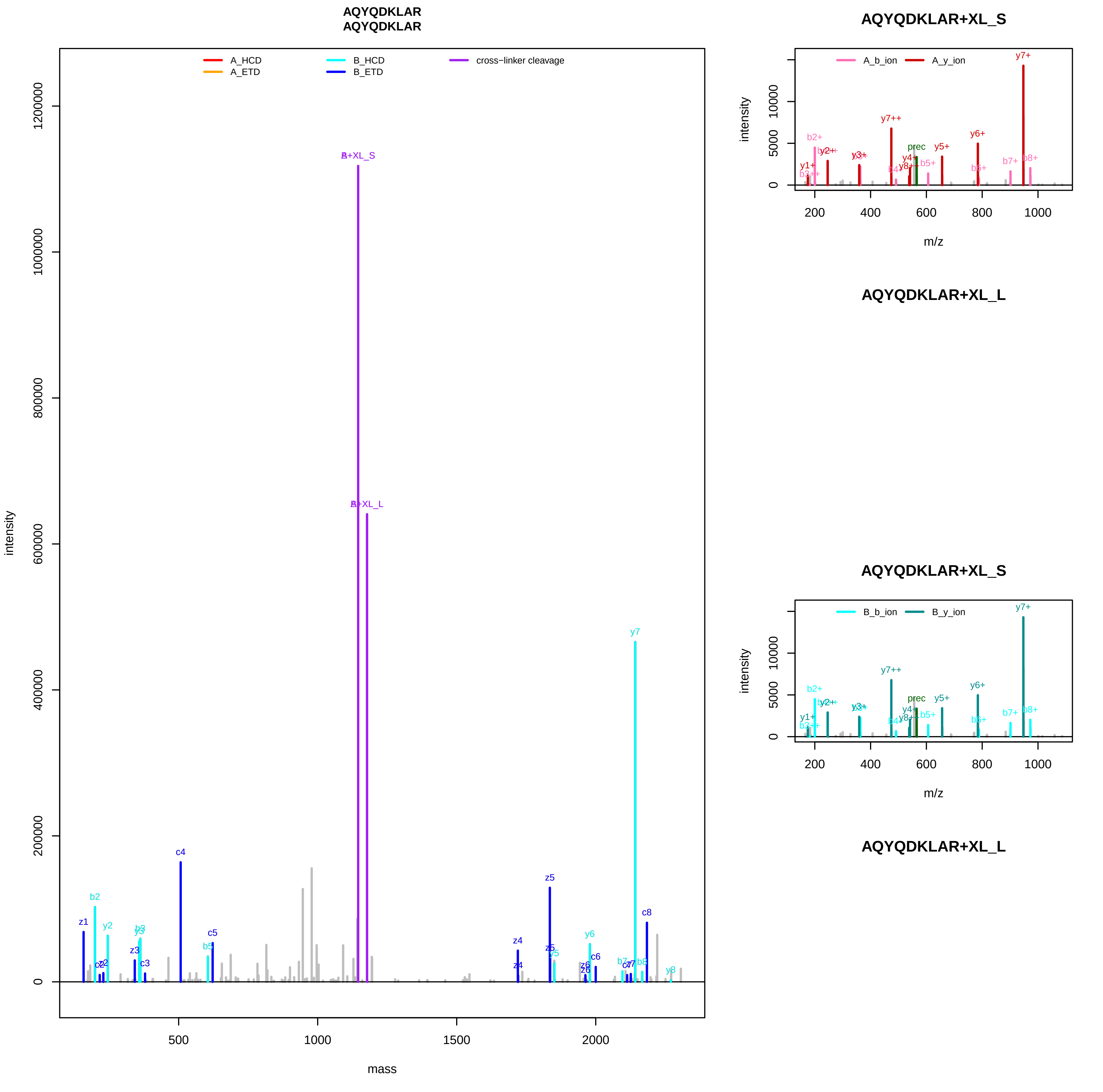
AQYQDKLAR
AQYQDKLAR
A_HCD
A_ETD
B_HCD
B_ETD
cross−linker cleavage
B+XL_S
B+XL_L
intensity
0
200000
400000
600000
800000
1000000
1200000
mass
500
1000
1500
2000
z1
b2
c2
z2
y2
b3
y3
z3
c3
c4
b5
c5
z4
z5
y5
z6
c6
y6
b7
c7
z7
b8
c8
y7
y8
AQYQDKLAR+XL_S
A_b_ion
A_y_ion
B_b_ion
B_y_ion
prec
b2+
b3++
y1+
y2+
y3+
y4+
y8++
b4+
b5+
y5+
y6+
b6+
b7+
b8+
y7++
y7+
m/z
200
400
600
800
1000
0
5000
10000
AQYQDKLAR+XL_L
